# Bites and Stings

**Published:** 1879-08

**Authors:** 


### BITES AND STINGS

are destructive from their acid nature; consequently the cure is an alkali. Pour a little spirits of hartshorn into a cup, wet a rag with it and bathe the wound ; it affords instant relief. Salaratus or soda will answer very well if you have no spirits of hartshorn.

A popular remedy for bites of poisonous snakes is to drink whiskey or any other spirits freely. As liquor enters the circulation immediately on being taken into the stomach, it mingles with the poison in the blood and destroys it. There is no doubt but this is the best remedy known; almost always preventing death from the poison, except in cases of persons who are hard drinkers: in these cases it is not reliable ; wash the wound with an alkali as above directed.

